# The Molecular Mechanism of Leptin Secretion and Expression Induced by Aristolochic Acid in Kidney Fibroblast

**DOI:** 10.1371/journal.pone.0016654

**Published:** 2011-02-03

**Authors:** Tsung-Chieh Lin, Tien-Chiang Lee, Shih-Lan Hsu, Chung-Shi Yang

**Affiliations:** 1 Graduate Institute of Life Sciences, National Defense Medical Center, Taipei, Taiwan, Republic of China; 2 Department of Education and Research, Taichung Veterans General Hospital, Taichung, Taiwan, Republic of China; 3 Center for Nanomedicine Research, National Health Research Institutes, Miaoli, Taiwan, Republic of China; Wellcome Trust Centre for Stem Cell Research, United Kingdom

## Abstract

**Background:**

Leptin is a peptide hormone playing pivotal role in regulating food intake and energy expenditure. Growing evidence has suggested the pro-inflammatory and fibrogenic properties of leptin. In addition, patients with renal fibrosis have higher level of plasma leptin, which was due to the increased leptin production. Aristolochic acid (AA) is a botanical toxin characterized to associate with the development of renal fibrosis including tubulointerstitial fibrosis. However, whether leptin is upregulated to participate in AA-induced kidney fibrosis remain completely unknown.

**Methodology/Principal Findings:**

In this study, leptin expression was increased by sublethal dose of AA in kidney fibroblast NRK49f determined by enzyme-linked immunosorbent assay and Western blot. Data from real-time reverse transcriptase-polymerase chain reaction revealed that leptin was upregulated by AA at transcriptional level. DNA binding activity of CCAAT enhancer binding protein α (C/EBP α), one of the transcription factors for leptin gene, was enhanced in DNA affinity precipitation assay and chromatin immunoprecipitation experiments. Knockdown of C/EBP α expression by small interfering RNA markedly reduced AA-induced leptin expression. Moreover, AA promoted Akt interaction with p-PDK1, and increased phosphorylated activation of Akt. Akt knockdown, and inhibition of Akt signaling by LY294002 and mTOR inhibitor rapamycin reduced leptin expression. Furthermore, treatment of LY294002 or rapamycin significantly suppressed AA-induced C/EBP α DNA-binding activity. These results suggest that Akt and C/EBP α activation were involved in AA-regulated leptin expression.

**Conclusions/Significance:**

Our findings demonstrate the first that AA could induce secretion and expression of fibrogenic leptin in kidney fibroblasts, which reveal potential involvement of leptin in the progression of kidney fibrosis in aristolochic acid nephropathy.

## Introduction

Leptin, an obese gene (ob) product initially identified in 1994, was named from the Greek word *leptos*, which means thin [1]. Leptin is a 16 kDa peptide hormone of cytokine family mainly secreted by adipocyte into blood stream and functions as a central mediator that negatively regulates satiety in hypothalamus, while its deficiency is associated with the development of obesity and metabolic syndrome [2]. However, the subsequent discovery of leptin receptor expression beyond brain tissue such as lung, liver and kidney implicates its action other than appetite regulation and energy metabolism [3], [4]. Growing evidences suggest the fibrogenic property of leptin. For example, mice receiving recombinant leptin enhanced fibrogenic response, while leptin receptor-deficient *fa/fa* rat and *db/db* mice are resistant to the development of liver fibrosis [5], [6]. The secretion of fibrogenic transforming growth factor-β (TGF-β) is enhanced by exogenous addition of leptin in cultured glomerular endothelial cells [7]. In rodent model of renal interstitial fibrosis, elevated TGF-β mRNA level, phosphorylated activation of Smad 2/3 and the up-regulated downstream target genes are significantly reduced in leptin deficient *ob/ob* mice [8]. Leptin was further considered as a cofactor of TGF-β activation, which enhanced TGF-β signaling in normal rat kidney fibroblasts [8]. These results implicate the regulatory role of leptin in renal interstitial fibrosis.

Aristolochic acid (AA) is a famous botanical toxin which has been characterized to associate with the development of aristolochic acid nephropathy (AAN). AAN, previously designated as Chinese-herb nephropathy (CHN), was originally reported in a group of women in Belgium who receiving sliming pills containing powdered root of Chinese-herb *Aristolochia fangchi* which is rich in AA [9]. These patients suffered from progressive interstitial fibrosis leading to end-stage renal disease, the stage that kidney permanently fails to work [9]. Besides, experimental AAN was also characterized to induce interstitial fibrosis after injection of AA to Wistar rats [10], [11].

Renal interstitial fibrosis is the process of renal fibroblasts activation and accumulation. The activated fibroblasts, myofibroblasts, are the main source of extracellular matrix deposition [12], [13]. Fibroblasts, a part of the interstitial connective tissue, are one of the important sources for cytokine synthesis and action which may result in fibrosis [14]. Since leptin belongs to a peptide hormone of cytokine family, and enhanced leptin expression by insulin stimulation has been reported in human skin fibroblasts [14]. In addition, patients with end-stage renal disease have higher level of plasma leptin, which was due to the increased leptin production [15]. Thus far, leptin has been considered to play an important role in progressive renal fibrosis. Renal fibroblast, in particular under the progression of fibrosis induced by AA, has not been intensively investigated with regard to biosynthesis and secretion of leptin. Therefore, the aim of this study is to explore the effect of AA on leptin production and to dissect the AA-induced downstream signaling *in vitro*. We found that AA could upregulate leptin in rat renal fibroblast NRK-49f cells through an Akt-C/EBP α signaling pathway. These findings imply the fibroblast-produced leptin maybe one of the factor which promotes the progression of renal fibrosis induced by AA.

## Results

### AA increased leptin expression in renal NRK-49f fibroblasts

**Figure 1:**
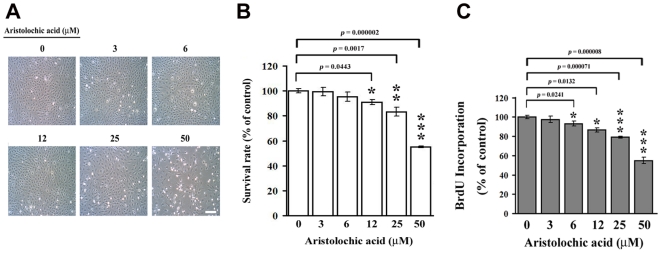
The effect of aristolochic acid on cell growth of NRK-49f cells. NRK-49f cells were treated with 0, 3, 6, 12, 25 and 50 µM of AA for 48 h. (A) Cell morphology was investigated by phase-contrast microscopy. Magnification 100X. Scale bar, 50 µM. (B) The cell survival rate was determined by MTT assay, and (C) the cell proliferation was examined by BrdU incorporation. Data are presented as mean±S.D. of 9 replicates from three independent experiments.

**Figure 2:**
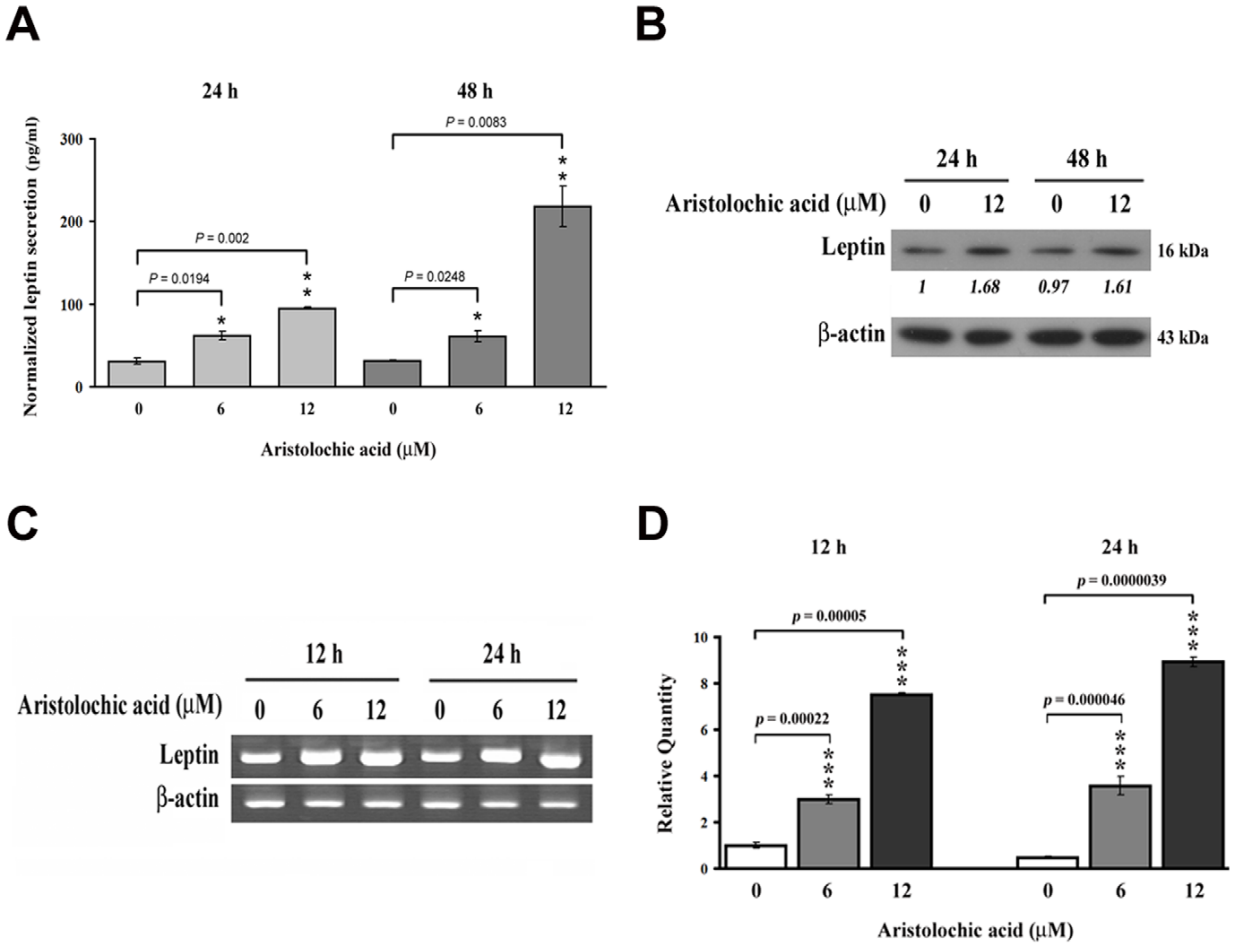
Aristolochic acid increased the expression and secretion of leptin. (A) Cells were treated with 0, 6 and 12 µM AA for 24 and 48 h. The level of secreted leptin was measured by ELISA. Data are presented as mean±S.D. of 9 replicates from three independent experiments. (B) Leptin expression was examined by Western Blot. NRK-49f kidney fibroblasts were treated without or with 12 µM AA for 24 and 48 h. (C) Expression of leptin mRNA. Fibroblasts were treated with 0, 6 and 12 µM AA for 12 and 24 h, the level of leptin mRNA was determined by RT-PCR and (D) real-time PCR.

Rat renal fibroblast NRK-49f cells were treated with different doses of AA for 48 h. As shown in Fig. 1A, low doses of AA (3∼12 µM) did not affect the growth of NRK-49f cells, whereas high dose of AA (50 µM) exhibited a strong antiproliferative effect, with approximately 50% growth inhibition, detected after 48 h incubation (Fig. 1B and 1C). Therefore, the low doses (≤12 µM) of AA were chosen for the following experiments of detecting cellular and molecular events. To examine whether AA affected leptin production, NRK-49f cells were treated without or with 6 and 12 µM AA for 24 and 48 h. Significant elevation of leptin secretion was observed in AA-treated cells compared to untreated controls, particularly at 48 h of 12 µM AA treatment (*p* = 0.0083) (Fig. 2A). Data from Western blot analysis showed that AA exposure led to an increase in the level of leptin protein in NRK-49 cells (Fig. 2B). To address whether the AA-induced leptin production might result from a transcriptional regulation, NRK-49f cells were stimulated without or with AA for 12 and 24 h, and the level of cellular leptin mRNA was measured by reverse transcriptase-polymerase chain reaction (RT-PCR) and real-time PCR (Fig. 2C & 2D). As presented in Fig. 2D, the amounts of leptin mRNA rose markedly after AA treatments. These results indicate that the leptin expression would be enhanced by AA at transcriptional level.

### The involvement of transcription factor C/EBP α in AA-induced leptin expression

**Figure 3:**
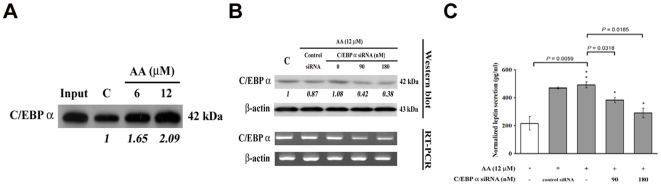
Involvement of C/EBP α in aristolochic acid-induced leptin expression. (A) Enhancement of C/EBP α DNA binding activity by AA. Fibroblasts were treated with 0, 6 and 12 µM AA for 3 h, nuclear extracts were isolated and C/EBP α DNA binding activity was analyzed by DAPA. (B) Knockdown of C/EBP α expression by siRNA. NRK-49f cells were transfected with 0, 90 and 180 nM of C/EBP α siRNA or control scramble siRNA for 8 h, and then cells were treated with 12 µM AA for another 48 h, the expression level of C/EBP α was examined by immunoblotting (upper panel) and RT-PCR (lower panel). (C) The secreted leptin was measured by ELISA. Data are presented as mean±S.D. of 9 replicates from three independent experiments.

C/EBP α has been reported as a key transcription factor implicated in the determination of the adipocyte differentiation which regulates leptin gene expression [16]. Therefore we examined the effects of AA on the activity and expression of C/EBP α. DNA affinity precipitation assay (DAPA) using nuclear extracts from NRK-49f cells and an oligonucleotide probe corresponding to a consensus binding site of C/EBP α generated a specific protein-oligonucleotide complex. As shown in Fig. 3A, the DNA binding activity of C/EBP α was enhanced dose-dependently upon AA treatment compared to controls. Nevertheless, the level of C/EBP α protein per se was not regulated by AA (Fig. 3B). Knockdown of C/EBP α expression with specific siRNA (Fig. 3B) suppressed AA-induced leptin secretion (Figs. 3C). These results suggested the involvement of C/EBP α in AA-mediated upregulation of leptin.

### Activation of PI3K-Akt signaling pathway by AA

**Figure 4:**
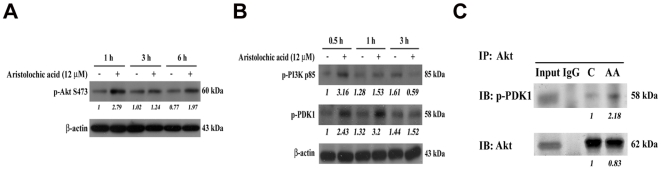
PI3K-Akt signaling pathway was activated upon AA treatment. (A) Examination of Akt activation. NRK-49f cells were incubated with 0 and 12 µM AA for 1, 3 and 6 h. The phosphorylated activation form of Akt was detected by immunoblotting. (B) The levels of phosphorylated PDK1 and PI3K-p85 were evaluated by Western Blot at indicated periods of AA treatment. (C) The interaction of phosph-PDK1 with Akt. After 1 h of 0 and 12 µM AA treatments, cells were immunoprecipitated with anti-Akt antibody. The immunoprecipitated pellets were further immnoblotted with anti-phospho PDK1 or anti-Akt antibody. The input and mouse IgG were served as positive and negative control, respectively.

Leptin production has been reported to be upregulated through PI3K-Akt-mTOR pathway in adipocytes [17]. To examine whether AA activated PKB/Akt, we performed Western blotting of lysates from untreated or AA-treated NRK-49f cells using antibodies against phospho-PKB/Akt. Elevated levels of Akt-Ser473 phosphoproteins were detected in AA-treated cells compared with control cultures (Fig.4A). Furthermore, the upstream molecules mediating Akt activation were proposed to be the phosphorylation of PI3K and PDK1 [18]. The phosphorylation of PDK1 and PI3K p85 respectively at Ser241 and Tyr458 are required for their kinase activity [19], [20], [21], which were also elevated upon AA stimulation (Fig. 4B). In addition, the interaction of p-PDK1 with Akt due to the phosphorylation activation by AA treatment was further confirmed by immunoprecipitation (IP) experiment (Fig. 4C), suggesting that AA triggered PI3K-Akt signaling activation in NRK-49f cells.

### Involvement of Akt and mTOR in AA-induced leptin expression

**Figure 5:**
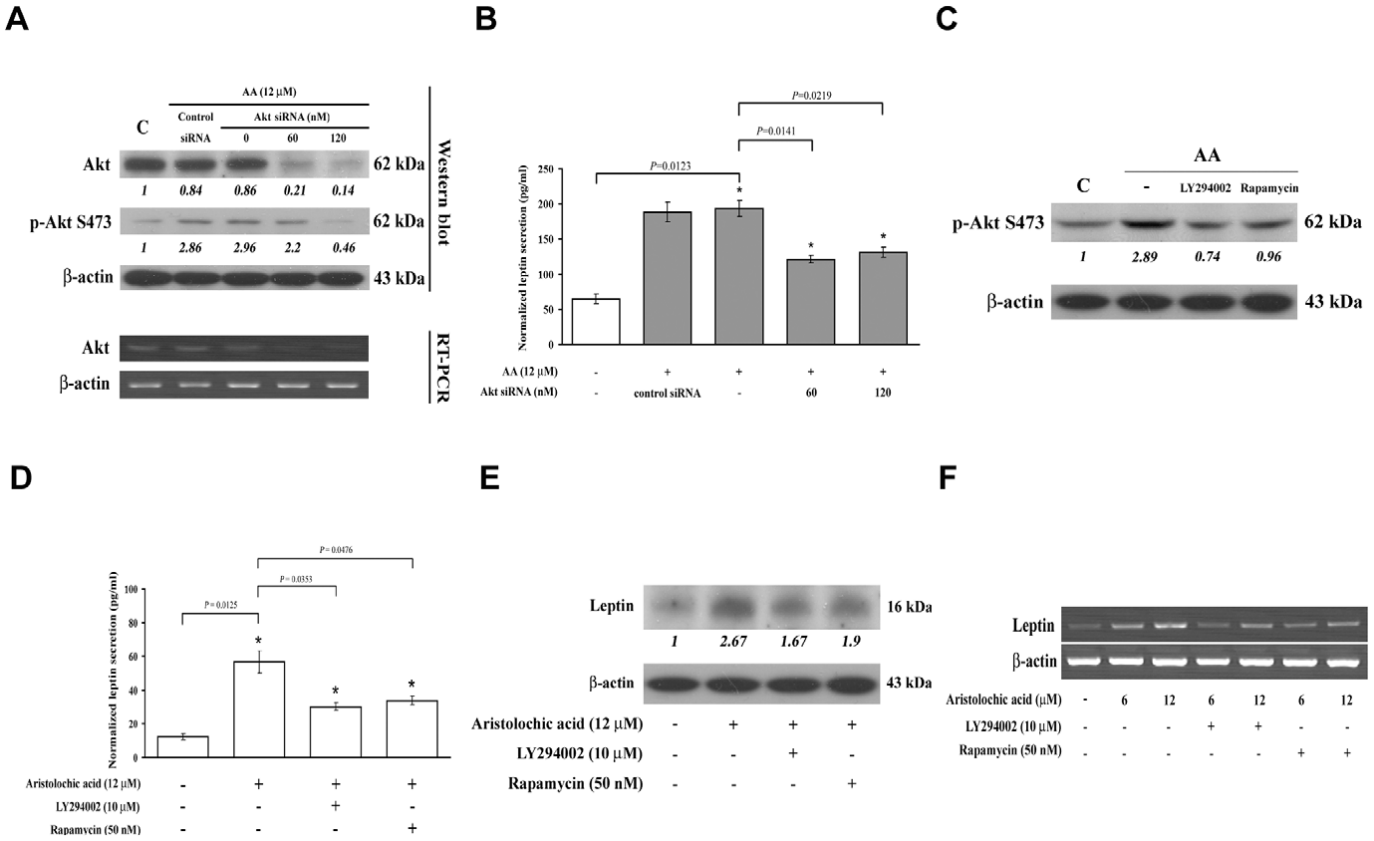
Involvement of PI3K-Akt signaling pathway in AA-induced elevation of leptin expression and C/EBP α DNA binding activity. (A) Knockdown of Akt by siRNA. Cells were transfected with 0, 60 and 120 nM of Akt siRNA or control scramble siRNA for 8 h, and then cells were treated with AA for another 48 h, the expression level of Akt was examined by immunoblotting (upper panel) and RT-PCR (lower panel). (B) Leptin secretion. Akt siRNA-transfected NRK-49f cells were treated with 12 µM AA for 48 h. The secreted leptin was measured by ELISA. (C) Analysis of Akt activation. Cells were pretreated with 10 µM LY294002 (PI3K inhibitor) or 50 nM rapamycin (mTOR inhibitor) 1 h prior 12 µM AA addition. After 3 h incubation, level of Akt phosphorylation was determined by immunoblotting. (D) Analysis of leptin expression. Cells were treated with PI3K-Akt signaling inhibitors, LY294002 or rapamycin for 1 h, and were followed by 48 h of AA administration. Level of leptin secretion and protein expression were measured by (D) ELISA and (E) immunoblotting, respectively. (F) Expression of leptin mRNA. After the 1 h pretreatment of indicated inhibitors, cells were then treated with AA for another 24 h. RNA was isolated, and the level of leptin mRNA was assessed by RT-PCR.

To assess the role of PI3K-Akt signaling in AA-induced leptin expression, endogenous Akt was knockdowned by specific siRNA, which reduced both cellular Akt expression and AA-induced Akt phosphorylation (Fig. 5A). Suppression of AA-induced leptin secretion was observed in Akt-knockdowned cells (Fig. 5B). Previous report indicates that leptin synthesis is positively regulated by mTOR which is a downstream target of Akt [22]. Hence, we further examined the role of mTOR in AA-mediated leptin expression. Rapamycin was reported to be an inhibitor of mTOR complex 1 (mTORC1) and even mTOR complex 2 (mTORC2), which are the downstream and upstream regulator of Akt respectively [18], [23], [24]. Here, we found that AA-induced phosphorylation of Akt was reduced by rapamycin, as well as LY294002 (Fig. 5C). Moreover, inhibition of AA-stimulated leptin secretion was observed by treatment of NRK-49f cells with LY294002 (*p* = 0.0353) or rapamycin (*p* = 0.0476) (Fig. 5D). In addition, AA-induced leptin expression was also abolished by the prior treatment of LY294002 or rapamycin (Fig. 5E). Furthermore, addition of LY294002 or rapamycin appeared to significantly inhibit the expression of leptin mRNA (Fig. 5F).

**Figure 6:**
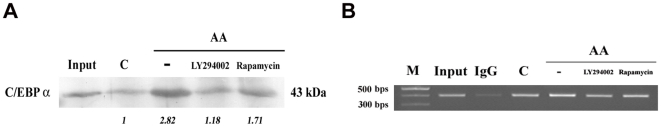
Involvement of PI3K-Akt signaling in the C/EBP α transactivation. Determination of C/EBP α DNA binding activity. After 1 h administration of LY294002 and rapamycin, NRK-49f cells were treated without or with 12 µM AA for 3 h. The DNA binding activity of C/EBP α was examined by (A) DAPA and (B) ChIP.

To address whether PI3K-Akt signaling pathway plays a role in C/EBP α transactivation upon AA treatment, NRK-49f cells were pretreated with LY294002 and rapamycin prior to AA. Addition of 10 µM LY294002 or 50 nM rapamycin abrogated AA-mediated C/EBP α DNA binding activity in the result of DAPA (Fig. 6A). Data of chromatin immunoprecipitation assay (ChIP) further confirmed that inhibition of PI3K-Akt signaling by LY294002 and rapamycin significantly inhibited intracellular C/EBP α-DNA binding induced by AA (Fig. 6B). These results showed AA-induced transactivation of C/EBP α was mediated via activation of the PI3K-Akt pathway.

## Discussion

AA, a nephrotoxic and carcinogenic plant alkaloid which is derived from *Aristolochia* species, could be a potential etiological factor for progressive renal interstitial fibrosis frequently associated with urothelial malignancies [25]. Renal interstitial fibrosis is the process of renal fibroblasts activation and accumulation. The activated fibroblasts, myofibroblasts, are the main source of extracellular matrix deposition. In particular, fibroblasts are one of the important sources for cytokine synthesis and action which may result in inflammation and fibrosis. However, renal fibroblasts have not been intensively investigated with regard to biosynthesis and secretion of leptin.

In this study, we found that the sublethal doses of AA increased the leptin expression in rat renal fibroblast NRK-49f cells at a transcriptional level. Leptin is a peptide hormone mainly produced by adipose tissue. However, recent evidence has shown that the placenta, skeletal muscle, and possibly stomach fundus are additional sites of leptin synthesis [26], [27]. Besides its effects on regulation of body weight, appetite and energy expenditure, leptin also exhibits influence on the immune system and may contribute to the deterioration of renal function. The peptide stimulates proliferation of glomerular endothelial cells, and increases TGF-β1 synthesis as well as collagen type IV production [7]. Leptin also increases collagen type I and surface TGF-β type II receptor synthesis in mesangial cells [28]. Infusion of leptin into normal rats fosters development of glomerulosclerosis and proteinuria [7]. In addition, leptin may stimulate profibrotic action in the kidney by sympathetic overactivity which has been associated with the progression of renal disease [29], [30]. These findings collectively suggest that the kidney is a target organ for leptin and that this peptide hormone might play an important role in renal pathophysiology. Our observations showed firstly that AA could upregulate the expression of the fibrosis-associated peptide hormone leptin in renal fibroblasts.

The regulation of leptin biosynthesis and secretion by AA in renal fibroblasts has not been studied yet. It has been reported that the promoter region of leptin gene contains transcription response elements including a TATA box, a CCAAT/enhancer binding protein (C/EBP) element, a leptin promoter specific factor (LP1) and a Sp1 site [31]. Point mutation of those conserved and functional regions respectively reduced promoter activity in rat adipocytes. In the results of both DAPA and ChIP, C/EBP α-DNA binding activity was induced by AA, whereas the assays using Sp1 and LP1 consensus binding sequences exhibited no obvious variation compared with the control (data not shown). C/EBP α is a basic region/leucine zipper transcription factor important for the transcription of most adipocyte genes and of other genes involved in energy metabolism [32]. Previous studies show that C/EBP α has been identified as a transactivator of the leptin promoter working through a consensus C/EBP binding site in the proximal leptin promoter [16], [33]. This site mediates activation of the leptin promoter by co-transfected C/EBP α in primary rat adipocytes and 3T3-L1 preadipocytes [16], [34]. Here, we showed that knockdown of C/EBP α by siRNA effectively reduced AA-induced leptin secretion, which indicated the involvement of C/EBP α in AA-regulated leptin expression in renal fibroblasts. The identification of C/EBP α in the upregulation of leptin expression induced by AA raises an obvious question. Does regulation of leptin expression by AA occur via modification, in amount or activity, of the C/EBP α that bind to leptin promoter? Our observations indicated that phosphorylation, but not expression, of C/EBP α is regulated by AA.

Several studies have demonstrated insulin-mediated increases in leptin gene transcription and synthesis [35], [36], [37]. Inhibitors of PI3K and MEK1/MEK2, as well as mTOR, had been showed to block insulin stimulated leptin release from isolated rat adipocytes [38]. A previous report indicated that leptin synthesis is regulated by PI3K, Akt and mTOR [22]. Here, we found that the leptin gene expression induced by AA in renal fibroblasts was regulated through activated PDK1, Akt and mTOR. However, the downstream signaling of mTOR that leads to a specific increase in leptin expression remains to be determined. AMP-activated protein kinase (AMPK) is an enzyme that works as a fuel gauge which becomes activated in situations of energy consumption. AMPK functions to restore cellular ATP levels by modifying diverse metabolic and cellular pathways [39]. Overexpressing dominant negative AMPK had been demonstrated to increase mTORC1 and leptin translation [40]. However, in this study, the level of phosphorylated activation of AMPK at T172 site was not affected by AA treatment (data not shown), suggesting that AMPK was not involved in regulation of leptin expression upon AA treatment in renal fibroblasts.

Our observations do not provide the direct evidence to demonstrate how AA transduced the downstream signaling to the cells. It has been reported that the uptake of AA is indicated by the anion transporter (OAT) in kidney proximal cells [41], [42]. AA exhibited high affinity to OAT1, as well as OAT3, while the OAT-mediated AA uptake was abolished in the presence of the OAT inhibitor probenecid [41], [42]. Hence, whether OATs exist and involve in the biological effects of AA in renal fibroblasts requires further investigation.

Taken together, our findings demonstrate that leptin synthesis and secretion were upregulated by AA through PI3K-Akt and C/EBP α activated pathway in renal fibroblasts, which might contribute to the progression of kidney fibrosis in aristolochic acid nephropathy.

## Materials and Methods

### Materials

1∶1 mixture of AA I and II and rapamycin were obtained from Calbiochem (CN Biosciences Notts, UK). 3-(4, 5-dimethylthiazol-2-yl)-2, 5-diphenyl tetrazolium bromide (MTT) and C/EBP α small interfering RNA (siRNA) were purchased from Sigma (St. Louis, MO, USA). Cell proliferation (BrdU) kit was purchased from Roche Applied Science (Mannheim, Germany). Rat leptin ELISA kit was purchased from Millipore (Bedford, MA). Anti-leptin antibody, anti β-actin antibody, anti-C/EBP α antibody, anti-Akt antibody and Akt siRNA were obtained from Santa Cruz (Santa Cruz, CA, USA). Anti-p-Akt^S473^ antibody, anti-p-Akt^T308^ antibody, anti-p-PDK1^S241^ antibody, anti-p-PI3K p85^Y458^ and LY294002 were purchased from cell signaling technology (Beverly, MA).

### Cell culture

Rat kidney fibroblast NRK-49f cell line was obtained from American Type Culture Collection. Cells were maintained in Dulbecco's modified Eagle's medium (DMEM) supplemented with 5% bovine serum (GIBCO), 1% non-essential amino acid, penicillin (100 unit/ml), and streptomycin (100 µg/ml). Bovine serum was reduced to 0.5% in the treatment of AA. Cells were incubated in 95% air, 5% CO_2_ humidified atmosphere at 37°C. AA was prepared with DMSO, and the control studies were performed using equivalent DMSO volume compared to the highest AA dose, which was calculated to be maximal 0.05% DMSO in the culture medium.

### Cell viability and BrdU incorporation assay

Cell viability and proliferation were assessed by the mitochondrial-dependent reduction of MTT to purple formazan and BrdU incorporation respectively. After culture of overnight in a 12-well plate, NRK-49f cells (1×10^4^ cells/well) were treated with 0, 3, 6, 12, 25 and 50 µM of AA. After 48 h treatment, MTT solution (100 µg/well) was added for another 2 h. The medium was removed and 200 µl of DMSO was added to each well and then vibrated for 10 min. Absorbance at 550 nm was measured using a microplate reader. The percentage of viable cells was calculated as follows: (absorbance of experimental group/absorbance of control group) x 100%. In the experiment of cell proliferation assay, 3×10^5^/well of cells seeded in 96-well plate were treated without or with AA for 48 h. BrdU incorporation and detection were in accordance with the manufacturer's instructions.

### Measurement of leptin secretion

Secreted leptin was collected from the 10 ml medium of 2–3×10^6^ cells, and was concentrated with Amicon Ultra-4 centrifugal filter devices (Millipore, Bedford, MA) at 4°C. The amount of leptin secretion into the culture medium was determined by enzyme-linked immunosorbent assay (ELISA) using a commercially available rat leptin ELISA kit (Millipore, Bedford, MA) according to the protocol recommended by the manufacturer. The secreted leptin level detected from each treatment had been normalized to 1×10^6^ cells in 1 ml medium.

### Western blot analysis

Cells were lysed at 4°C in RIPA buffer containing 50 mM Tris-HCl (pH 7.4), 150 mM NaCl, 1% Triton X-100, 0.25% Sodium deoxycholate, 5 mM EDTA (pH 8.0), and 1 mM EGTA and supplemented with protease and phosphatase inhibitors. After 20 min of lysis on ice, cell debris was removed by microcentrifugation, followed by quick freezing of the supernatants. The protein concentration was determined by Bradford method. In our experiments, 25–50 µg of protein was loaded. In particular for the phosphorylated protein detection, 100 µg of protein was loaded. Equal amounts of proteins were separated by SDS-polyacrylamide gels and then electrophoretically transferred from the gel onto a PVDF membrane (Millipore, Bedford, MA). After blocking with 5% non-fat milk, the membrane was reacted with specific primary antibodies overnight at 4°C and then incubated with horseradish peroxidase conjugated secondary antibody for 1 h. The blots were visualized using ECL-Plus detection kit (PerkinElmer Life Sciences, Inc. Boston, MA, USA). The blot images were quantitated by densitometry using the GelPro analysis software and normalized with the internal control (β-actin).

### Semi-quantitative RT-PCR and real-time PCR

Total cellular RNA was extracted by RNA-Bee™ RNA isolation kit (TEL-TEST, Friendswood, TX) in accordance with the manufacturer's instructions. One microgram of total RNA was reverse-transcribed using Advantage RT for PCR Kit (Clontech, Mountain View, CA) at 42°C for 1 h as described in the manufacturer's protocol. PCR conditions for rat leptin were 94°C for 5 min and 37 cycles at 94°C for 30 s, 56°C for 30 s and 72°C for 60 s, followed by a final extension step at 72°C for 5 min by Bio-Rad icycle (Bio-Rad). Primer sequences were as follows: rat leptin: 5′-CCAGGATGACACCAAAACCC-3′ (sense) and 5′-TCCAACTGTTGAAGAATGTCC-3′ (antisense) with product size of 405 bp; rat β-actin: 5′- TCTACAATGAGCTGCGTGTG-3′ (sense) and 5′-GGTCAGGATCTTCATGAGGT-3′ (antisense) with product size of 314 bp. For each combination of primers, the kinetics of PCR amplification was studied. The number of cycles corresponding to plateau was determined and PCR was performed at exponential range. PCR products were then electrophoresed through a 1% agarose gel and visualized by ethidium bromide staining in UV irradiation. The mRNA levels were also determined by real-time PCR with ABI PRISM 7900 Sequence Detector system according to the manufacturer's instructions. β-actin was used as endogenous control. PCR reaction mixture contained the SYBR PCR master mix (Applied Biosystems), cDNA, and the primers. Relative gene expression level (the amount of target, normalized to endogenous control gene) was calculated using the comparative Ct method formula E^-ΔΔCt^. The primer sequences were as follows: rat leptin: 5′-CTGTGGCTTTGGTCCTATCT-3′ (sense) and 5′-TCCATCTTGGACAAACTCAG-3′ (antisense).

### Small interfering RNA (siRNA) transfection

Cells were seeded in 6-well plates and transfected with Akt or C/EBP α specific siRNA using Lipofectamine 2000 (Invitorgen) in accordance with the manufacturer's instructions. After 6 h of incubation, the medium was replaced with complete medium. Rat C/EBP α (SASI_Rn01_00034372) and Akt siRNA (SASI_Rn01_00063656) were purchased from sigma, and control siRNA (sc-37007) was purchased from Santa Cruz. siRNA sequences were as follows: rat C/EBP α: 5′-GCCUGAGAGCUCCUUGGUC-3′ (sense) and 5′-GACCAAGGAGCUCUCAGGC-3′ (antisense); rat Akt1: 5′-GGCACAUCAAGAUAACGGA-3′ (sense) and 5′-UCCGUUAUCUUGAUGUGCC-3′ (antisense). Sequences of control siRNA were not released from the manufacturer.

### Immunoprecipitation

Cells were washed with PBS. The lysate was prepared by adding 1 ml of immunoprecipitation assay buffer (50 mM Tris-HCl, pH 7.8, 150 mM NaCl, 5 mM EDTA, 0.5% Triton X-100, 0.5% Nonidet P-40, 0.1% deoxycholate, and 10 µg/ml each of leupeptin, aprotinin, and 4-(2-aminoethyl)benzenesulfonyl fluoride) to the cells. Then the lysate was centrifuged using a microcentrifuge at 10,000 rpm for 20 min. The supernatant was precleaned with Protein A/G Plus-Agarose beads (Santa Cruz Biotechnology, Santa Cruz, CA, USA), and followed centrifugation. Anti-Akt antibody was added to the supernatant at 4°C for overnight. Protein-A/G-agarose beads were added to the lysate, and the mixture was incubated with shaking for 1 h at 4°C. The beads were finally collected by centrifugation and washed three times with immunoprecipitation assay buffer. Proteins binding to the beads were eluted by adding 20 µl of 2X electrophoresis sample buffer and analyzed by immunoblotting with anti-phospho PDK1 antibody.

### DNA affinity precipitation assay (DAPA)

DNA affinity precipitation assay was carried out by immobilizing 1 µg of the biotinylated probe per sample onto strepatvidin-agarose beads (Invitrogen), as recommended by the manufacturer. Nuclear extracts from indicated time points and treatments were precleaned with streptavidin-agarose beads for 1 h, with gentle rotation at 4°C. After centrifugation, the supernatants were incubated with biotinylated probe in binding buffer [150 mM KCl, 12 mM Hepes (pH = 7.9), 4 mM Tris–HCl (pH = 7.9), 12% (v/v) glycerol, 1 mM EDTA, 1 mM dithiothreitol] for overnight. Streptavidin-agarose beads were added, and incubated for 1 h with gentle rotation at 4°C. The precipitated DNA-protein complexes were then washed three times with binding buffer, resolved on SDS-polyacrylamide gel electrophoresis, and detected by western blot using anti-C/EBP α specific antibody.

### Chromatin immunoprecipitation (ChIP) assay

NRK-49f cells were subjected to various treatments as indicated in the figure. The cells were then fixed with 1% formaldehyde at 37°C for 10 min. Cells were collected by centrifugation in PBS containing protease inhibitors and were lysed in SDS-lysis buffer followed by the ChIP assay as described previously [43]. Immunoprecipitation was performed overnight with antibodies against C/EBP α. After immunoprecipitation, 60 µl of salmon sperm DNA-protein A agarose was added for 1 h at 4°C to capture the immune complexes. The agarose beads were washed, chromatin extracted and protein-DNA cross-links reversed. DNA was purified by DNA clean-up purification Kit (Promega) and was analyzed by RT-PCR analysis. The primer sequences were as follows: 5′-GATTACCCGGCTCATACCAA-3′ (sense) and 5′-GCACCAAGCTGTCCACACTA-3′ (antisense) with product size of 381 bp.

### Statistical analysis

All data are presented as mean±S.D. Statistical analysis used Student's *t*-test for pairs with the following significance levels: * *P*<0.05, ** *P*<0.01, *** *P*<0.001. All figures were generated from at least three repeated experiments with similar patterns.
